# Toward standardized environmental detection of antibiotics and ARGs for regulatory interpretation and concern tiering

**DOI:** 10.3389/fmicb.2026.1868218

**Published:** 2026-06-05

**Authors:** Ningning Pi, Xiaoyao He, Juan Zhang, Lu Zhu, Xiaoyan Wu, Lili Yang, Yi Li, Rong Xiang, Xuan Wu

**Affiliations:** 1 Key Laboratory of Environmental Chemistry and Ecotoxicology of Organic Pollutants of Chongqing, Chongqing Ecological and Environment Monitoring Center, Chongqing, China; 2 Yangtze River Upstream Ecological Barrier Research Think Tank, Chongqing Ecological Environment Monitoring Center, Chongqing, China; 3 Precision Medicine Center, The Second Affiliated Hospital of Chongqing Medical University, Chongqing, China; 4 Key Laboratory of Clinical Laboratory Diagnostics (Ministry of Education), College of Laboratory Medicine, Chongqing Medical University, Chongqing, China; 5 Western (Chongqing) Institute for Digital-Intelligent Medicine, Chongqing National Biomedicine Industry Park, Chongqing, China

**Keywords:** antibiotic exposure detection, antimicrobial resistance diagnostics, ARG detection, biosensors, environmental antimicrobial resistance detection, regulatory interpretation

## Abstract

Environmental detection of antimicrobial resistance has expanded rapidly, but many programs still treat detection of antibiotic resistance genes (ARGs) or resistant bacteria as the primary endpoint. As a result, outputs are often only weakly linked to antibiotic exposure conditions and remain difficult to compare, interpret, and use consistently for follow-up decisions. This review examines how detection of antibiotics and ARG/ARB can be standardized for regulatory interpretation and qualitative concern tiering. It defines standardization across pre-analytical, analytical, data, and interpretive layers; proposes a minimum detection package; and describes how comparison-ready chemical and biological outputs can support integrated interpretation. It also reviews chromatographic, sequencing, biosensor, and other recognition-based platforms, arguing that platform choice should match screening, confirmation, quantification, or discovery roles. This review then outlines a four-tier framework for concern and decision priority, together with reporting and implementation elements needed to translate detection results into proportionate follow-up. Environmental AMR detection becomes more useful when antibiotic exposure, ARG/ARB evidence, and contextual information are standardized and interpreted together rather than handled as separate streams.

## Highlights

- Antibiotic and ARG/ARB detection should be interpreted together rather than as separate evidence streams.
- Reference antibiotic detection and rapid screening methods should be assigned explicit roles.
- A minimum detection package should include purpose, context, antibiotic exposure, and ARG/ARB evidence.
- Comparison-ready chemical and biological outputs are prerequisites for consistent tiered interpretation.
- Concern tiers and structured reporting can link detection results to proportionate follow-up.

## 1 Introduction

Environmental surveillance of antimicrobial resistance has expanded rapidly, yet much of the field still treats detection as the primary endpoint (Berendonk et al., 2015; Larsson and Flach, 2022; Samreen et al., 2021). Across wastewater, surface waters, and related compartments, studies commonly report the presence, abundance, or temporal variation of antibiotic resistance genes (ARGs), resistant bacteria, or both (Wellington et al., 2013; Karkman et al., 2018; Pazda et al., 2019). Such outputs are valuable for characterizing occurrence and identifying potential hotspots, but they do not in themselves provide a sufficient basis for management or regulatory use. A surveillance signal may indicate that resistance determinants are present, but not whether the observed finding reflects routine background, elevated concern, conditions plausibly consistent with antibiotic selection pressure, or a situation that warrants prioritized follow-up (Bengtsson-Palme et al., 2023; Liguori et al., 2022; Hart et al., 2023; Larsson et al., 2023). In this review, detection refers to the measurement or recognition of antibiotics, ARGs, or ARB in environmental samples; monitoring refers to planned or repeated detection within a defined sampling design; and surveillance refers to the programmatic use of monitoring outputs for comparison, interpretation, tier assignment, and follow-up decisions.

Recent literature has already addressed many parts of this problem. Framework- and policy-oriented reviews have clarified why environmental antimicrobial resistance should be monitored, which implementation questions remain unresolved, and which data gaps continue to constrain the use of surveillance outputs (Bengtsson-Palme et al., 2023; Hart et al., 2023; Pruden et al., 2021; Punch et al., 2025). Reviews that consider antibiotics and ARGs together have likewise pushed the field beyond occurrence data alone (Nava et al., 2022). Wastewater-focused syntheses have highlighted interpretive limits, inconsistent target selection, and weak links between monitoring outputs and downstream use (Chau et al., 2022; Tiwari et al., 2022). Standardization-focused papers have shown that surveillance results are highly sensitive to differences in sampling design, laboratory practice, target selection, and data handling (Berendonk et al., 2015; Liguori et al., 2022; Anjum et al., 2021). In parallel, work on environmental antibiotic concentrations has shown that exposure data can be difficult to generate and report in fully comparable forms, yet remain central to judging whether observed resistance signals arise under plausible selective pressure (Bengtsson-Palme et al., 2023; Murray et al., 2021; Hanna et al., 2023b; Fabregat-Safont et al., 2023; Magee et al., 2023; Khan et al., 2017). Although other co-selective pressures may also shape environmental resistance patterns in specific settings, antibiotic exposure remains the most direct and policy-relevant chemical counterpart to ARG/ARB surveillance. The highest-overlap recent papers are summarized in Table 1, with the broader screened mapping provided in Supplementary Table S1. Taken together, these studies show that the field does not lack conceptual attention; the more pressing issue is how surveillance outputs can be converted into comparable evidence for repeated interpretation and follow-up decisions (Bengtsson-Palme et al., 2023; de Beltran Heredia et al., 2025; Gholipour et al., 2024).

**Table 1:** Positioning summary of selected recent review and framework papers most closely aligned with the present review.

Reference	Main focus of prior review/Framework	Closest overlap with this review	Key gap left unresolved
de Beltran Heredia et al. (2025)	Practical framework for environmental antibiotic resistance monitoring in freshwater ecosystems	Closest recent monitoring-framework paper in spirit; practical implementation framing	Does not make antibiotic exposure detection a co-required pillar and does not provide a harmonized decision-tier architecture
Bengtsson-Palme et al. (2023)	Why environmental AMR monitoring is needed, how it could be implemented, and what data are needed	Agenda-setting review on environmental AMR monitoring design and implementation	Identifies exposure-data needs but does not translate them into a standardized co-monitoring requirement with explicit interpretive tiers
Liguori et al. (2022)	Standardized methods and quality control for AMR monitoring in water environments	Core benchmark for ARG/ARB detection standardization and QA/QC	Strong on methods and QC, but not organized around antibiotic exposure detection plus harmonized regulatory tiering
Hart et al. (2023)	Environmental AMR surveillance from a national regulator perspective	Most directly regulator-oriented overlap paper in the recent literature	Frames policy questions well, but does not operationalize antibiotic co-monitoring or a repeatable concern-tier system

AMR, antimicrobial resistance; ARG, antibiotic resistance gene; ARB, antibiotic-resistant bacteria; QA/QC, quality assurance/quality control. The broader screening-based mapping is provided separately as Supplementary Table S1.

**Figure 1:**
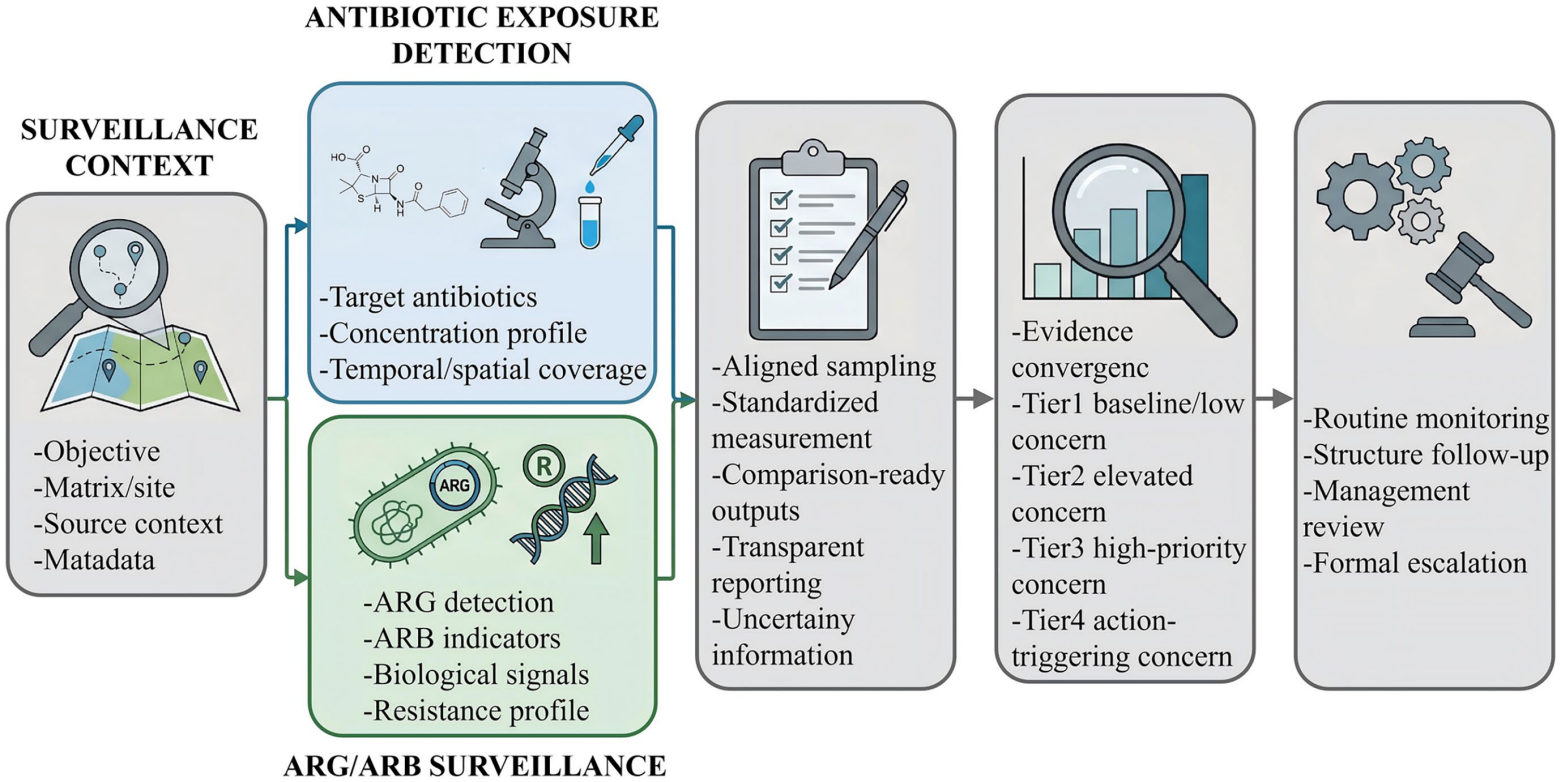
Surveillance-to-decision framework for standardized environmental detection of antibiotics and ARGs. The framework links surveillance context, antibiotic exposure detection, and ARG/ARB detection to standardized outputs, integrated interpretation, concern tiering, and follow-up decisions. The two evidence streams are shown as complementary inputs that should be aligned and interpreted together rather than reported as separate endpoints. ARG, antibiotic resistance gene; ARB, antibiotic-resistant bacteria.

Against this background, this review asks how environmental surveillance of antibiotics and ARGs can be standardized so that results are more comparable, easier to interpret, and more usable in decision-making. It addresses three linked questions: what evidence should be included in a minimum monitoring package, how antibiotic exposure and ARG/ARB measurements should be standardized across the surveillance workflow, and how these outputs can be translated into harmonized tiers of concern for follow-up and management decisions. The aim is not to derive direct measures of environmental or public-health risk from routine surveillance, but to organize sufficiently comparable and convergent evidence for prioritization. The specific added value of this review is therefore to argue that chemical exposure standardization and biological resistance standardization should not be developed as parallel checklists. Instead, they should be designed from the outset for joint interpretation, so that antibiotic measurements, ARG/ARB evidence, and contextual information can be translated into comparable concern tiers and follow-up decisions (Figure 1).

## 2 Persistent standardization gaps across environmental detection workflows

In antibiotic-resistance detection, prior reviews have already shown that standardization in environmental AMR work is not only a bench-level issue (Bengtsson-Palme et al., 2023; Chau et al., 2022; de Beltran Heredia et al., 2025). For regulatory interpretation, however, the problem still has to be treated as a workflow issue rather than mainly as a laboratory-method and QC issue. A result may be methodologically rigorous and still be poorly suited to comparison or judgment if sampling design is unclear, metadata are incomplete, reporting formats are non-comparable, or the basis for classification remains implicit. In this review, standardization is therefore treated as a multi-layer property of integrated antibiotic-resistance surveillance: it concerns how antibiotic exposure and ARG/ARB evidence are co-designed, how each stream is measured, how outputs are processed and reported, and how they are ultimately translated into statements of concern or priority (Berendonk et al., 2015; Bengtsson-Palme et al., 2023; Liguori et al., 2022; Pruden et al., 2021; Bengtsson-Palme et al., 2018).

This emphasis is especially important for antibiotics because their interpretive logic differs from that of many other environmental contaminants. For numerous pollutants, concentration is read primarily against direct toxicological or ecological endpoints. By contrast, one of the most consequential environmental hazards of antibiotics is their capacity to select for and enrich resistance in microbial communities. Concentration data therefore are not a sufficient endpoint on their own: if surveillance is meant to judge resistance-related concern, antibiotic measurements need to be interpreted together with resistance evidence rather than in isolation (Liguori et al., 2022; Fabregat-Safont et al., 2023; Magee et al., 2023; Angeles and Aga, 2018; Knight et al., 2024).

First, pre-analytical standardization concerns how the antibiotic-resistance surveillance question is framed before any measurement is generated. This includes the surveillance objective, matrix selection, site-selection logic, sampling timing and frequency, sample handling, preservation, transport, and the contextual metadata recorded alongside each sample. In an integrated antibiotics-ARG framework, it also includes whether the design can meaningfully relate the chemical and biological signals. A program designed to characterize antibiotic occurrence alone, one designed to assess whether exposure conditions are plausibly consistent with selection, and one designed to evaluate whether an intervention changes both antibiotic and ARG/ARB signals may each generate valid data, but their outputs are not automatically interpretable on the same basis unless the design logic is explicit (Hart et al., 2023; de Beltran Heredia et al., 2025). Different surveillance objectives will still require different field designs. The standardization requirement is that design choices and metadata are explicit enough that differences in either the exposure or resistance layer can be interpreted rather than merely noted.

Second, analytical standardization concerns how the antibiotic and resistance pillars are measured once the surveillance design has been defined. On the antibiotic-exposure side of the framework, this includes analyte-selection logic, extraction and preparation choices, detection and quantification limits, calibration, quality assurance and quality control, matrix-effect management, and the treatment of censored results. On the ARG/ARB side, it includes target selection, assay- or sequencing-level quality controls, quantification conventions, and performance reporting. The chemical and biological pillars do not require identical methods, nor should they, because they address different targets, operate under different technical constraints, and often rely on different measurement platforms. They do, however, require shared minimum expectations for transparency, performance, and comparability. In this framework, comparability is not only a within-stream issue but also a between-stream issue, because antibiotic and ARG/ARB results are later interpreted together. Sections 4–6 make this more concrete through aligned measurement and reporting expectations, tiered interpretation, and decision-use outputs. Without those minimum expectations, surveillance programs may generate values that are internally valid yet difficult to compare across laboratories, sites, time series, or evidence streams (Liguori et al., 2022; Fabregat-Safont et al., 2023; Magee et al., 2023; Angeles and Aga, 2018; Knight et al., 2024).

Third, data standardization concerns how raw measurements are converted into usable outputs. For antibiotics, it includes consistent use of units, explicit handling of non-detects, and transparent derivation of summary values. For ARG/ARB surveillance, it includes reporting conventions, normalization choices, denominators, and the metadata needed to determine whether observed differences reflect environmental variation or differences in processing and representation. This layer matters because the challenge is not only whether each stream is internally comparable, but also whether antibiotic and resistance data can be read together without ambiguity. Two programs may report the same compound or gene target, yet use different sample bases, aggregation rules, or reporting denominators, thereby producing outputs that are not directly interchangeable (Knight et al., 2024; Huijbers et al., 2020; Munk et al., 2022). A decision-oriented review of antibiotic-resistance surveillance must therefore treat data formatting and reporting conventions as integral to standardization rather than as editorial detail.

Fourth, interpretive standardization concerns how combined chemical and biological outputs are converted into comparable judgments. This layer is often the least developed, yet it is the one most directly linked to environmental management. It asks how surveillance outputs are translated into statements such as low concern, elevated concern, or priority for follow-up; what uncertainty should accompany those statements; and what minimum evidence pattern is required before stronger conclusions are drawn (Hart et al., 2023; Murray et al., 2021). This step is especially important in antibiotic-resistance surveillance because antibiotic concentrations do not map onto concern in the same direct way as many conventional contaminant endpoints. Instead, interpretation depends on whether exposure evidence, resistance evidence, and contextual information converge strongly enough to justify a given level of concern. Standardization therefore remains incomplete when measurements are reproducible but still weakly connected to judgment. It becomes complete only when comparable antibiotic and ARG/ARB evidence can also be translated into comparable conclusions.

**Figure 2:**
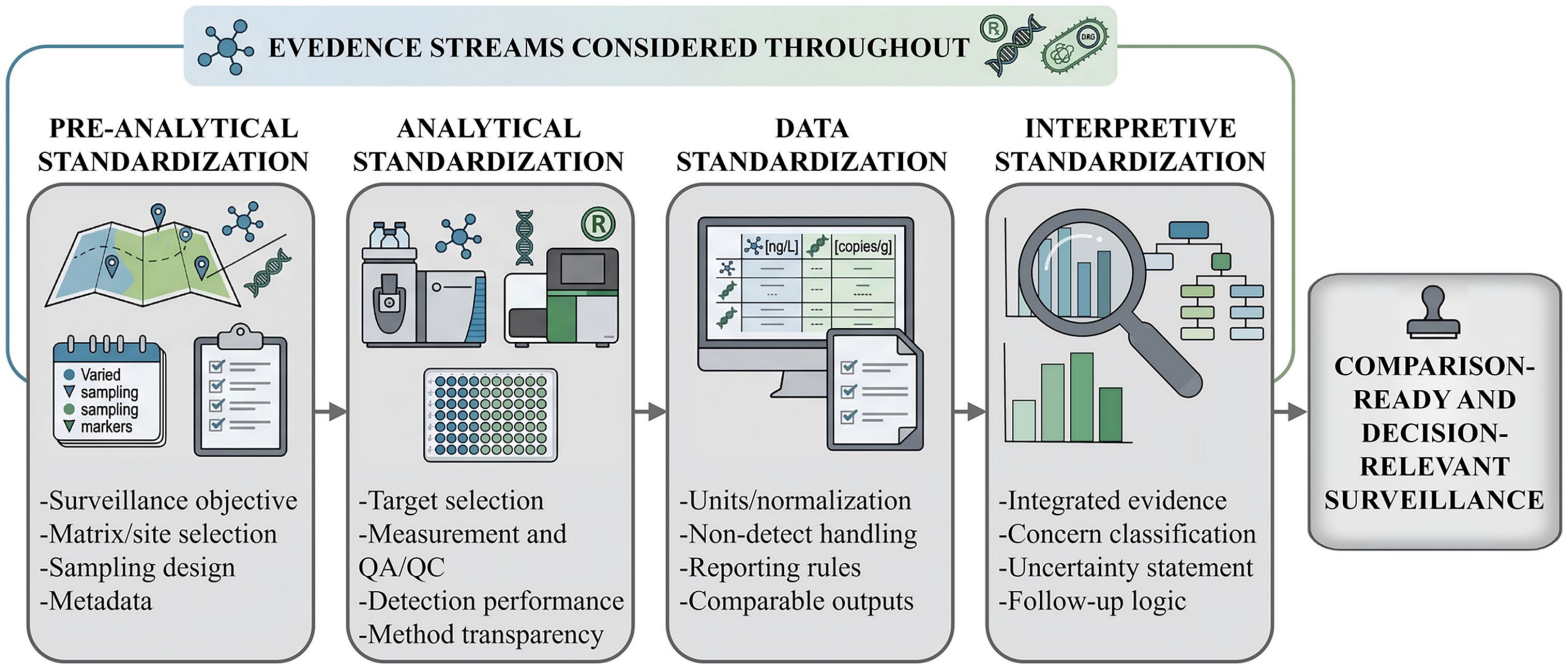
Persistent standardization gaps across environmental detection workflows. Standardization is organized across four workflow layers: pre-analytical design, analytical measurement, data processing, and interpretation. Antibiotic exposure and ARG/ARB evidence need to remain traceable across these layers to generate comparison-ready and decision-relevant surveillance outputs.

These layers are analytically separable but operationally linked, and the remainder of the review follows this workflow logic. Section 3 translates pre-analytical design into a minimum detection package by defining the purpose, exposure layer, resistance layer, and contextual metadata needed before interpretation is possible. Section 4 then addresses analytical and data-level standardization by specifying how antibiotic exposure and ARG/ARB detection outputs should be generated, reported, and aligned. Sections 5 and 6 build on these comparison-ready outputs to address interpretive standardization, tier assignment, decision-facing reporting, and follow-up use. Accordingly, Figure 2 summarizes how workflow-level standardization connects detection design, measurement alignment, data comparability, and regulatory interpretation.

## 3 Defining the minimum detection package

Once standardization is treated as a workflow property, the next question is what evidence must be present before interpretation is possible. ARG or ARB detection alone may be adequate for exploratory screening, but it is not sufficient when surveillance is expected to support comparison, prioritization, or escalation. A minimum detection package is therefore needed to define the smallest evidence set capable of supporting interpretable surveillance outputs (Berendonk et al., 2015; Larsson et al., 2023; Pruden et al., 2021).

**Figure 3:**
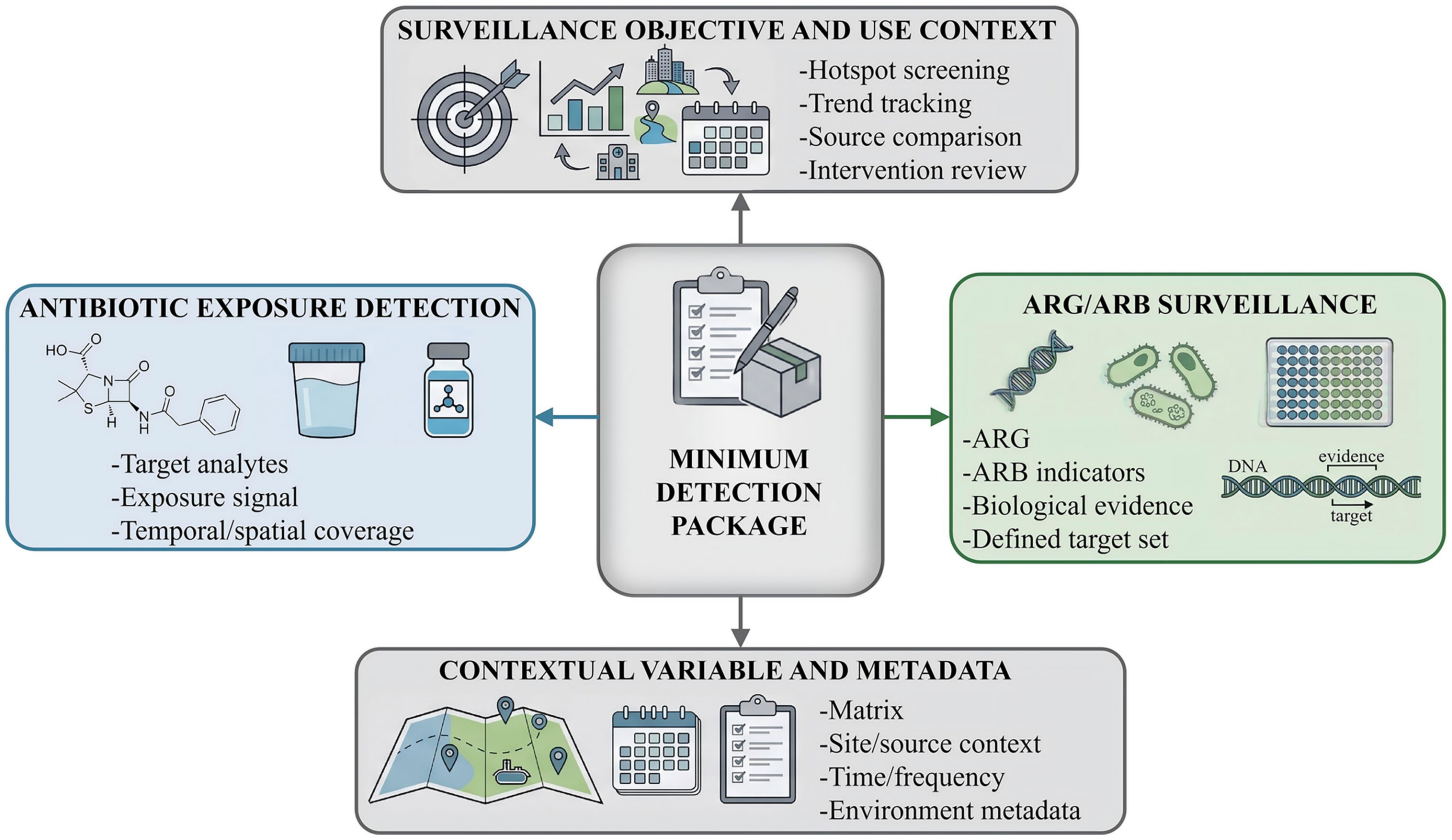
Minimum detection package for interpretable environmental surveillance. The minimum package includes four core elements: surveillance objective and use context, antibiotic exposure detection, ARG/ARB detection, and contextual variables with metadata. Together, these elements define the basic evidence structure needed before surveillance outputs can support comparative interpretation.

In this review, the minimum package comprises four elements: a defined surveillance objective and use context, an antibiotic exposure layer, an ARG/ARB surveillance layer, and the contextual metadata needed to interpret both. These elements are deliberately minimal rather than exhaustive. Their purpose is to identify what should be treated as irreducible if antibiotics are to be interpreted as resistance-relevant pollutants rather than as chemicals assessed in isolation. Figure 3 summarizes this package conceptually.

### 3.1 Surveillance objectives and use contexts

The minimum package begins with an explicit statement of surveillance purpose because design intent determines what later comparisons can legitimately support. Monitoring undertaken for hotspot screening, temporal trend tracking, source comparison, intervention evaluation, or permit and management review does not ask the same question, even when the same matrices are sampled. Without a defined use context, readers cannot know what kind of inference the dataset was designed to support (Bengtsson-Palme et al., 2023; Hart et al., 2023; de Beltran Heredia et al., 2025).

### 3.2 Antibiotic exposure detection as a required package element

Antibiotic exposure monitoring is a required component of the minimum package because, without an exposure layer, resistance surveillance cannot distinguish mere occurrence from a finding plausibly linked to ongoing or recent selective pressure. Recent reviews and risk-assessment papers have already made this need visible, even if concentration data remain difficult to generate and compare (Murray et al., 2021; Hanna et al., 2023b; Khan et al., 2017; Hanna et al., 2023a; Bengtsson-Palme and Larsson, 2016). This prioritization does not imply that other co-selective pressures are unimportant. Rather, antibiotic exposure is treated here as the minimum chemical evidence layer because it is the most direct and policy-relevant counterpart to antibiotic resistance surveillance, whereas other stressors are better handled as context-dependent extensions when the surveillance objective requires them. For that reason, antibiotic monitoring should not be treated as an optional enhancement reserved for technically ambitious studies.

At the minimum-package level, this does not require a comprehensive screen of every antibiotic of possible relevance. Rather, it requires a justified set of target analytes appropriate to the surveillance objective and source context, together with enough temporal and spatial coverage to read the exposure signal against the biological signal. The precise panel can vary by matrix, source type, and intended use, but the package should include at least some direct information on antibiotic concentrations rather than relying solely on inference from source category or land use.

### 3.3 ARG/ARB detection package

The third element is ARG/ARB detection itself. Its precise composition may vary by surveillance context, but the minimum package should still contain a defined biological evidence set rather than opportunistic reporting of whatever targets happen to be measured. At minimum, this evidence set should specify which ARGs, ARG classes, resistant-bacteria indicators, or combined outputs are being used as the biological basis for interpretation, together with enough information to clarify what the measurements are intended to represent. For targeted methods such as PCR, qPCR, or targeted sequencing panels, the ARG set should cover the antibiotic classes highlighted by exposure detection, so that the chemical and biological evidence streams remain directly comparable (Pazda et al., 2019; Anjum et al., 2021; Knight et al., 2024; Huijbers et al., 2020). The point at this stage is not to prescribe a universal target list, but to avoid detection outputs that remain too loosely defined for comparison.

This element belongs in the minimum package because antibiotic exposure data alone cannot establish whether resistance-related signals are absent, stable, or elevated in the monitored setting. If exposure monitoring indicates whether selection is plausible, the biological block indicates whether a resistance signal requiring interpretation is actually present. The biological pillar is therefore indispensable, but it is not self-sufficient. Its role in the minimum package is to keep the framework grounded in direct resistance surveillance while avoiding the mistaken assumption that biological detection alone resolves interpretive questions.

### 3.4 Contextual variables and metadata

The final element of the minimum package is contextual metadata. Measurements that lack core contextual information are difficult to compare and often difficult to interpret. At minimum, the package should record the environmental matrix sampled, site type, source category or likely influence, sampling time and frequency, and relevant treatment, discharge, or receiving-environment context (Cacace et al., 2019; Quintela-Baluja et al., 2019; Rodriguez-Mozaz et al., 2015). Depending on the objective, additional variables such as hydrological conditions, season, or basic physicochemical descriptors may also be necessary. The point is not to create an exhaustive ecological characterization, but to ensure that surveillance outputs do not become isolated values detached from their environmental setting.

This element belongs in the package because both chemical and biological measurements are interpretable only in context. A concentration measured in influent, effluent, a downstream reach, sediment, or reclaimed water does not carry the same implication. Nor does the same ARG abundance mean the same thing across all matrices or surveillance designs, particularly when fecal loading, source influence, and receiving-environment conditions differ (Karkman et al., 2019). Contextual metadata therefore provide the interpretive frame within which the other evidence blocks can be compared.

Taken together, these four elements define the minimum detection package proposed in this review: an explicit surveillance objective, an antibiotic exposure detection layer, an ARG/ARB detection layer, and the contextual metadata needed to interpret them. The point of this section is to define what must be present before surveillance outputs can plausibly support comparative interpretation. The next question is how the antibiotic exposure component should be standardized and integrated with ARG/ARB surveillance.

## 4 Standardizing antibiotic exposure and ARG/ARB detection for integrated interpretation

Previous reviews have already documented many of the methodological challenges and cross-study comparability problems affecting both antibiotic monitoring and ARG/ARB surveillance (Liguori et al., 2022; Hart et al., 2023; Hanna et al., 2023b; Fabregat-Safont et al., 2023; Magee et al., 2023; de Beltran Heredia et al., 2025; Hanna et al., 2023a). This section therefore focuses on how antibiotic exposure and ARG/ARB outputs should be made interpretable on the same basis. In this framework, antibiotic exposure helps judge whether conditions are plausibly consistent with selection, whereas ARG/ARB surveillance shows whether a resistance signal requiring interpretation is actually present. Read together, the two streams also support target prioritization: biological evidence can indicate which antibiotic classes or source contexts warrant closer chemical attention, while exposure evidence helps judge the significance of observed resistance patterns (Hanna et al., 2023a).

The aim is to produce comparison-ready outputs within each stream and then align them where interpretation depends on their convergence. For antibiotics, this means transparent analyte selection, sampling alignment, and exposure summaries that can be read against the biological window. For ARG/ARB evidence, it means explicit target or method choice, quantitative basis, normalization or annotation rules where relevant, and outputs that can be compared within and across programs.

### 4.1 Standardizing antibiotic exposure detection

Standardizing antibiotic exposure detection begins once a minimum analyte panel has been justified at the package-design stage. Earlier reviews have already detailed the analytical challenges associated with matrix effects, analyte instability, extraction choices, and detection limits (Fabregat-Safont et al., 2023; Magee et al., 2023; Thurner and Alatraktchi, 2023). The issue here is how to produce exposure outputs that remain interpretable when later read alongside ARG/ARB evidence. At minimum, programs should state why particular antibiotic classes were selected, whether measurements represent grab samples, composites, or aggregated periods, how chemical sampling was aligned with the biological monitoring window, how non-detects and censored values were handled, and which comparison frame is being used, such as site baseline, upstream-downstream contrast, pre/post intervention, or source-receiver pairing (Magee et al., 2023). Stable panel logic over time is especially important if longitudinal or cross-site comparison is intended (Byrnes et al., 2025).

For integrated interpretation, exposure outputs should do more than list concentrations. They should summarize whether each monitored antibiotic signal is absent, intermittent, persistent, or elevated relative to the declared comparison frame, and they should distinguish isolated detections from repeated findings that overlap the biological sampling window (Byrnes et al., 2025). Where several antibiotics are monitored, reporting should also show which classes dominate the exposure profile, because that is the level at which later comparison with targeted ARG or ARB evidence becomes most interpretable (Hanna et al., 2023b). Without that structure, chemical data may remain analytically valid but still be difficult to read against the resistance signal.

### 4.2 Standardizing ARG/ARB detection

The complementary task on the biological side is to ensure that ARG/ARB detection outputs remain comparison-ready and interpretable in the same framework. Prior reviews have already discussed methodological diversity and comparability challenges in ARG/ARB monitoring. These approaches support different levels of inference and should therefore be standardized according to their intended surveillance role rather than presented as interchangeable options (Liguori et al., 2022; Chau et al., 2022; Tiwari et al., 2022; de Beltran Heredia et al., 2025). The practical question here is what must be declared for biological evidence to be read on the same basis as the exposure layer (Knight et al., 2024). At minimum, programs should make explicit the biological evidence block being used, such as sentinel ARGs, resistant-bacteria indicators, broader resistance classes, or sequencing-derived resistome summaries, and the interpretive role that evidence is intended to play. If phenotypic indicators are used, the organism, phenotype definition, and counting basis should be explicit (Flach et al., 2021); if targeted gene assays are used, the selected ARGs should match the antibiotic classes prioritized in the exposure layer (Knight et al., 2024); if sequencing-based outputs are used, the summary unit and intended comparison level should be stated upfront (Munk et al., 2022).

Once the biological approach has been chosen, outputs should be expressed with a clear quantitative basis, declared denominator, and transparent normalization or annotation rules where relevant. For targeted measurements, this means stating whether results are reported per volume, per biomass proxy, or as resistant fractions, how normalization was performed, and whether non-detects or low-count results were censored (Knight et al., 2024). For sequencing-based outputs, it means declaring the reference database and version, the annotation and filtering thresholds, and whether interpretation is based on counts, relative abundance, or broader resistome summaries (Liguori et al., 2022; de Beltran Heredia et al., 2025; Bengtsson-Palme and Larsson, 2016; Cacace et al., 2019; Quintela-Baluja et al., 2019). This is especially important because ARG databases differ in curation scope, resistance-gene definitions, and classification structure, so database choice can change the resulting ARG profile and its downstream interpretation (Papp and Solymosi, 2022; Alcock et al., 2020; Doster et al., 2020; Feldgarden et al., 2022). Without that information, nominally similar biological results may not support the same comparison or the same kind of inference across programs.

Phenotypic and genotypic evidence are not interchangeable, but they can be complementary if their inferential role is declared upfront. What matters for integrated surveillance is that the chosen biological stream be stable enough to support repeated comparison and explicit enough to show whether it is being used as a marker of general resistance burden, of specific clinically relevant determinants, or of change over time (Cacace et al., 2019; Flach et al., 2021).

### 4.3 Detection platforms and recognition strategies

Detection platforms should be standardized according to their surveillance function rather than treated as interchangeable technologies. Platform choice affects not only analytical performance, such as sensitivity, specificity, speed, and matrix tolerance, but also the type of interpretation that can be supported, including preliminary screening, confirmatory measurement, targeted quantification, or profile-level discovery. In an integrated antibiotic-ARG framework, the key question is therefore not only what a platform can detect, but whether its output is fit for the intended interpretive role.

For antibiotic exposure monitoring, LC–MS/MS and related chromatographic mass-spectrometry methods should be treated as the quantitative and reference-oriented detection approaches. Their major strength is the ability to provide compound-specific concentration estimates across multiple antibiotic classes, supported by calibration, matrix-effect assessment, detection and quantification limits, and quality-control procedures. These features make LC–MS/MS most appropriate for confirmatory measurement, trend analysis, and comparison with exposure-based interpretation frameworks (Fabregat-Safont et al., 2023; Magee et al., 2023; Angeles and Aga, 2018; Byrnes et al., 2025).

By contrast, immunoassays and recognition-driven biosensor formats, including aptamer-based, electrochemical, optical, and SERS-related platforms, are better positioned as screening or rapid-recognition tools. They may be useful for field-deployable detection, preliminary prioritization, or high-frequency monitoring, but their outputs should be interpreted cautiously unless selectivity, dynamic range, matrix tolerance, calibration transfer, and false-positive or false-negative behavior have been evaluated in the relevant environmental matrix (Frigoli et al., 2024; Pan et al., 2024; Mehlhorn et al., 2018; Magnano San Lio et al., 2023).

For ARG and ARB surveillance, the same function-based logic should be applied. Culture-based phenotypic detection provides information on viable resistant bacteria and can support phenotypic confirmation, but its interpretation depends on the organism selected, antibiotic breakpoint or concentration used, counting basis, and culture conditions (Chau et al., 2022; Tiwari et al., 2022; Flach et al., 2021). qPCR, digital PCR, and HT-qPCR are most useful as targeted quantification tools when the surveillance objective requires repeated measurement of predefined genes, resistance classes, or sentinel targets. Their strength lies in sensitivity, quantitative comparability, and suitability for longitudinal or source-comparison designs, provided that primer/probe sets, standards, inhibition controls, limits of detection, and normalization denominators are clearly reported (Liguori et al., 2022; Knight et al., 2024). Metagenomic sequencing, in contrast, is better understood as a profile-level and discovery-oriented approach. It can characterize broader resistome composition, detect non-targeted resistance determinants, and support comparison of ARG profiles across sites or countries, but its outputs are strongly shaped by DNA extraction, sequencing depth, database choice, annotation thresholds, and abundance-normalization rules (de Beltran Heredia et al., 2025; Munk et al., 2022; Papp and Solymosi, 2022; Alcock et al., 2020; Doster et al., 2020; Feldgarden et al., 2022).

Emerging methods such as CRISPR/Cas assays, isothermal amplification, and biosensor-based AMR detection may help bridge targeted recognition and rapid deployment, especially when short time-to-result or multiplexed detection is needed (Kao and Alocilja, 2025; Elbehiry et al., 2025; Phan et al., 2022). However, in regulatory-facing environmental surveillance, novelty alone should not determine platform choice. A practical workflow may use biosensors or immunoassays for rapid screening, LC–MS/MS for quantitative antibiotic confirmation, qPCR or dPCR for targeted ARG quantification, and metagenomics for broader resistome profiling or discovery. Standardization should therefore specify the intended functional role of each platform and the minimum performance evidence required before its outputs are combined with antibiotic exposure and ARG/ARB data for tiered interpretation.

### 4.4 Integrating the two detection streams for interpretation

The central standardization problem is not simply whether the chemical and biological streams are each internally comparable, but whether they can be interpreted together on a common basis. Integration does not mean collapsing antibiotics and ARG/ARB evidence into a single metric. It means aligning them through shared sampling logic, compatible comparison frames, and explicit rules for how exposure modifies the reading of resistance evidence and how resistance evidence, in turn, helps guide later target prioritization.

In practice, this requires at least four forms of alignment: temporal alignment between chemical and biological sampling; contextual alignment so that both streams are interpreted against the same site and source setting; comparison alignment so that each stream is read against an explicit baseline or reference frame; and reporting alignment so that exposure and biological outputs can be juxtaposed without ambiguity. When these conditions are met, antibiotic detection can indicate whether selection is plausible, ARG/ARB detection can indicate whether a resistance signal requiring interpretation is present, and the two together can generate a more decision-relevant statement than either stream can support alone.

This integrated logic also explains why joint standardization is necessary. Antibiotic data help interpret the formation, maintenance, or propagation of resistance signals, whereas ARG/ARB data help evaluate the resistance relevance of antibiotic contamination. The biological stream may also feed back into chemical prioritization by indicating which resistance classes, source settings, or clinically important determinants warrant closer antibiotic-target coverage in subsequent monitoring rounds. Once both streams have been made comparison-ready and interpretively aligned, the next step is to translate their convergence into harmonized concern tiers.

## 5 A harmonized concern-tiering framework for regulatory interpretation

**Figure 4:**
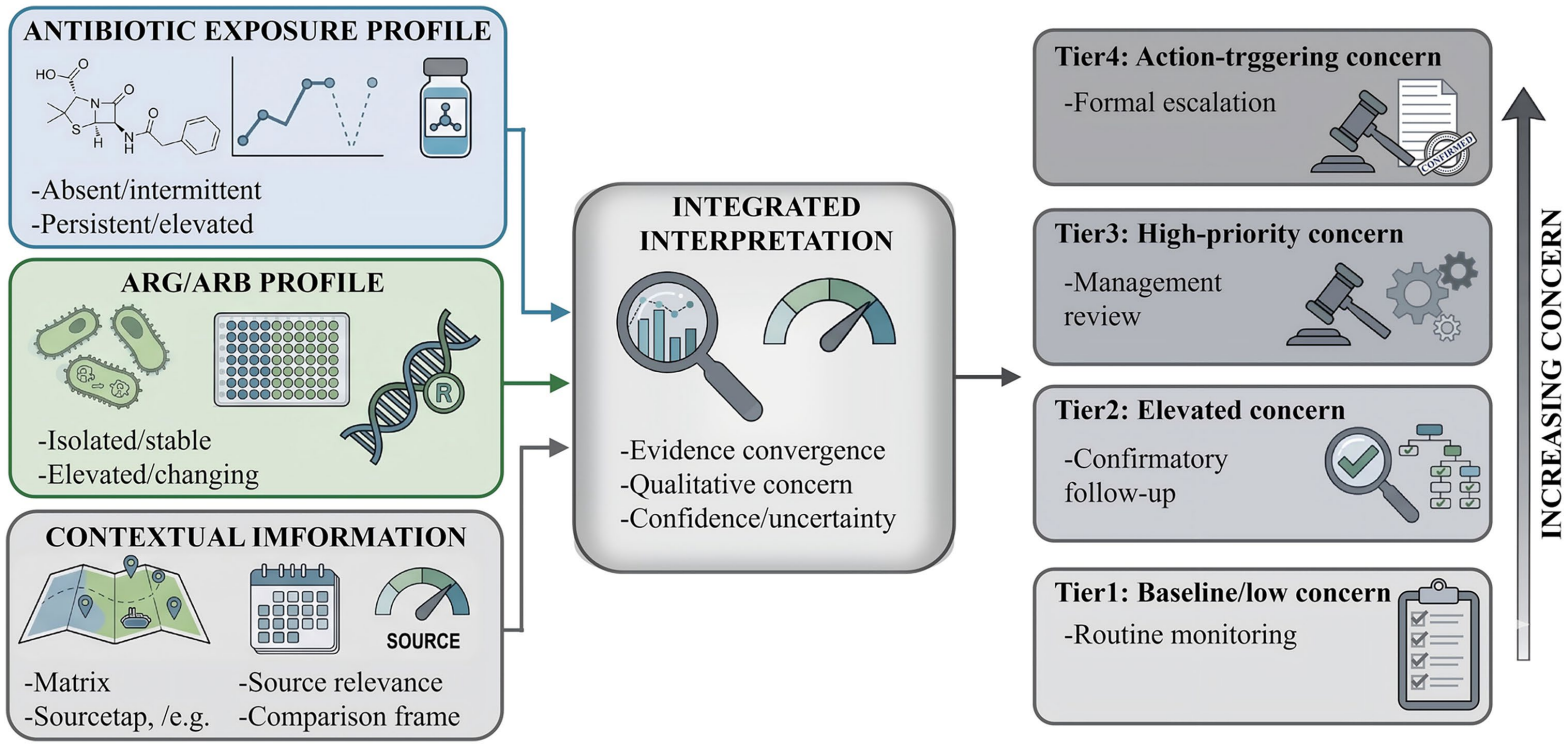
Harmonized qualitative concern-tiering framework for regulatory interpretation. Antibiotic exposure profiles, ARG/ARB profiles, and contextual information are integrated to assign qualitative concern tiers. The tiers represent increasing decision priority and follow-up intensity, rather than direct quantitative estimates of environmental or public-health risk.

Once antibiotic exposure and ARG/ARB evidence have been made comparison-ready, the next step is to interpret them together with contextual information so that surveillance findings can support prioritization. The tiers proposed here should therefore be understood as tiers of surveillance concern and decision priority, not as direct measures of environmental or public-health risk. Because the available evidence does not support universal numerical cutoffs across all matrices and use contexts, the framework is intentionally semi-structured rather than threshold-based and should not be interpreted as a formal risk-assessment scheme. Figure 4 shows the overall logic.

### 5.1 Evidence used for tier assignment

Tier assignment should be based on three evidence components: the antibiotic exposure profile, the ARG/ARB profile, and the contextual information needed to interpret both. Exposure data indicate whether antibiotic signals are absent, intermittent, persistent, or elevated. ARG/ARB data indicate whether resistance-related signals are isolated, stable, elevated, or changing relative to the comparison frame. Context determines whether those signals arise in a setting where comparison is meaningful. None of these components is sufficient on its own, so tiering should be based on their combined pattern rather than on any single result viewed in isolation.

Accordingly, detection alone should not be treated as enough for a high-concern classification. A site with repeated ARG/ARB elevation but poorly characterized antibiotic exposure may justify closer review, but it should not be interpreted in the same way as a site where elevated resistance signals coincide with persistent antibiotic exposure in a clearly relevant source context.

### 5.2 Tier definitions and uncertainty

For practical use, the framework can be organized into four tiers. Tier 1 represents baseline detection or low concern. It applies when antibiotic and/or ARG/ARB signals are low, sporadic, weakly supported, or difficult to interpret because comparability is limited. In such cases, the result supports routine observation rather than escalation.

Tier 2 represents elevated concern. It applies when one or more evidence components depart from baseline clearly enough to warrant attention, but the overall picture remains incomplete. This may include repeated antibiotic detection without clearly elevated resistance signals, persistent biological elevation with incomplete exposure characterization, or a site where the comparison frame is meaningful but uncertainty remains substantial. Such results support closer scrutiny and confirmatory follow-up.

Tier 3 represents high-priority concern. It applies when antibiotic exposure and ARG/ARB signals converge more clearly, for example when persistent or elevated antibiotic exposure coincides with persistent or elevated resistance signals in a relevant source or receiving-environment context. This tier does not require proof of downstream health impact. Rather, it indicates that the surveillance pattern is coherent enough to rank above routine findings in management review and prioritization.

Tier 4 represents action-triggering concern. It should be used only when the combined evidence is strong enough to justify formal escalation. In practice, this means repeated or persistent concern across the chemical and biological evidence streams, data quality strong enough to rule out obvious methodological artifact as the main explanation, and a context in which management or regulatory response is plausible. This tier does not by itself establish legal non-compliance or quantify public-health risk, but it does indicate that the surveillance record is strong enough to trigger formal follow-up.

Each tier should be reported together with an uncertainty statement. At minimum, this should indicate whether confidence in the classification is provisional, moderate, or strong, based on data completeness, comparability, temporal coverage, and contextual sufficiency (Bengtsson-Palme et al., 2023; Liguori et al., 2022). This is important because the same nominal tier should not carry the same weight when one dataset is short, incomplete, or poorly aligned and another is repeated, internally consistent, and well contextualized (Hart et al., 2023).

### 5.3 Tier-to-action mapping and management relevance

The practical value of tiering lies in linking different levels of concern to different levels of follow-up. Tier 1 generally supports continued routine monitoring. Tier 2 supports structured follow-up, such as confirmatory resampling, improved alignment of chemical and biological sampling, or focused source characterization (Flach et al., 2021). Tier 3 supports directed management review, including evaluation of likely sources, treatment or discharge performance, spatial persistence, and possible intervention options. Tier 4 supports formal escalation within the relevant management or regulatory system, which may include intensified monitoring, source-control review, treatment-performance assessment, or broader environmental management action (Byrnes et al., 2025).

This framework should support prioritization without inflating weak evidence. Sparse, poorly comparable, or context-poor datasets should not be translated into strong claims of risk. Its value lies in linking standardized monitoring outputs to proportionate follow-up.

In this sense, concern tiering is the interpretive bridge between comparison-ready detection outputs and decision use. Once antibiotic exposure and ARG/ARB evidence have been read together and assigned to a concern tier, the next task is to report those judgments in a form that can be used within surveillance programs.

## 6 Reporting templates, decision interfaces, and implementation in detection programs

**Figure 5:**
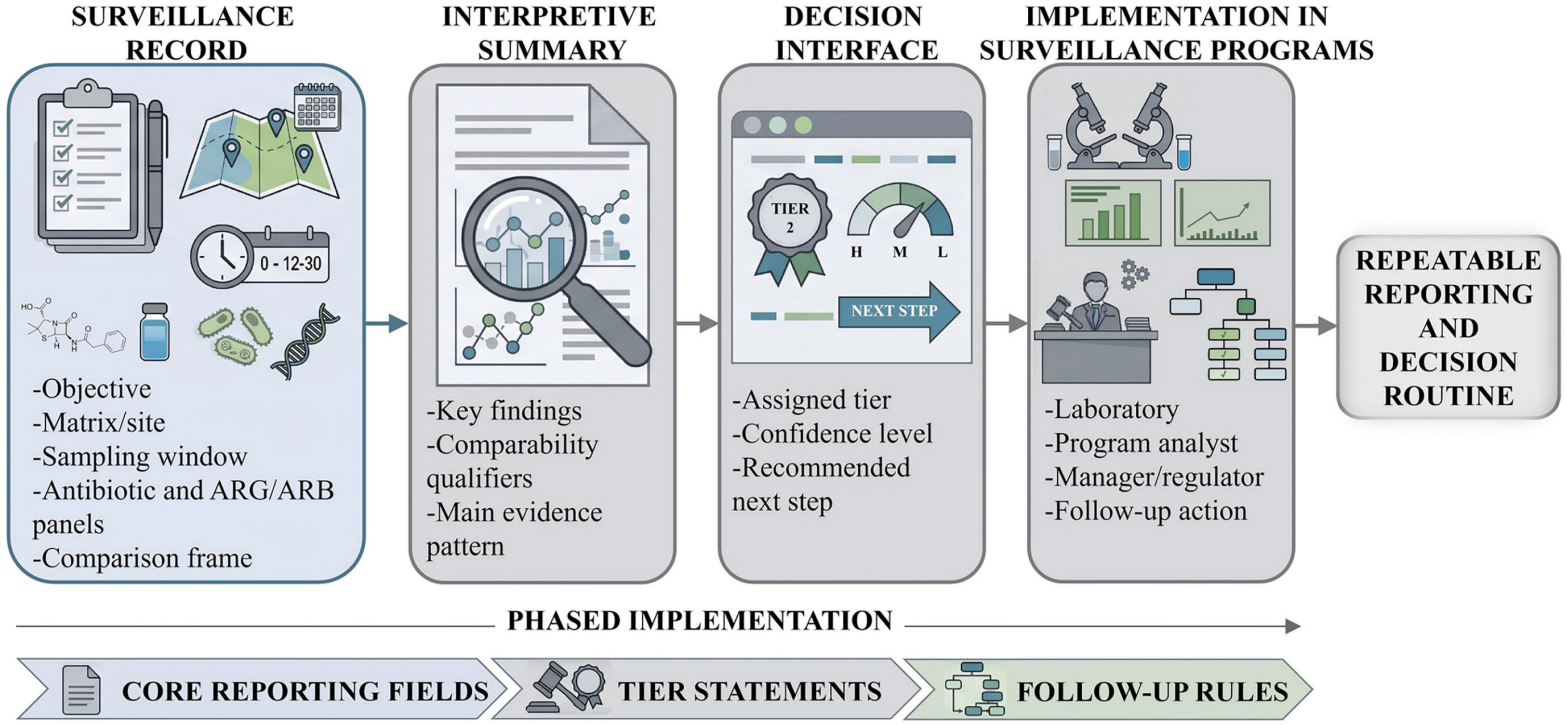
Decision-oriented reporting and implementation workflow. Standardized detection outputs are translated within surveillance programs into a detection-output record, interpretive summary, decision interface, and implementation workflow.

This section considers how aligned antibiotic and ARG/ARB outputs should be reported so they can be used in prioritization and follow-up. The reporting layer should convert comparison-ready detection outputs into a decision-facing format that shows what was found, how it was interpreted, how much confidence that interpretation deserves, and what follow-up is recommended (Bengtsson-Palme et al., 2023; Hart et al., 2023). Figure 5 shows that workflow.

### 6.1 Core elements of a decision-oriented report

A decision-oriented report should separate the detection-output record, the interpretive summary, and the decision handoff (Babu Rajendran et al., 2023). At minimum, it should identify the surveillance objective, matrix and site type, sampling window, antibiotic and ARG/ARB panels, declared comparison frame, key comparability qualifiers, assigned tier, uncertainty level, and recommended next step, because the meaning of a surveillance classification depends on what it is being compared against (Chau et al., 2022; de Beltran Heredia et al., 2025).

The comparison frame should be explicit, whether it is a site-specific baseline, an upstream-downstream contrast, or a program-level reference condition (Price et al., 2024). Without that frame, even a formally assigned tier is difficult to interpret consistently across rounds, sites, or users.

The report should also state why the tier was assigned. A high-priority or action-triggering result should not appear as a label alone. It should show, in brief, what pattern of antibiotic evidence, ARG/ARB evidence, and contextual information supported that classification, and where important uncertainty remains.

### 6.2 Presenting tiered results for decision use

The decision interface should be simple and direct (Delpy et al., 2024). Each result should include a short tier statement, a confidence statement, and a follow-up category. For example, routine monitoring, structured follow-up, directed management review, and formal escalation should be presented as clearly different outcomes.

The interface should also indicate whether interpretation is limited by incomplete sampling, changed methods, weak comparability, or short time series (Lappan et al., 2024). A short note is usually enough. For example: “Tier 2, moderate confidence; follow-up recommended because antibiotic detections were repeated, but the time series remains short.” The point is to make the detection output usable without forcing the reader to reconstruct the logic from raw tables or figures.

### 6.3 Stepwise implementation in surveillance programs

In practice, this framework is unlikely to be adopted all at once. A more realistic approach is phased implementation. Programs can begin by standardizing the minimum detection package and core reporting fields, then add tier statements and structured follow-up rules once the evidence base becomes stable enough to support them (Hart et al., 2023; de Beltran Heredia et al., 2025).

A practical early step is to harmonize reporting across repeated detection cycles within surveillance programs. Even before full inter-program standardization is achieved, programs can improve usability by reporting the same core fields, using the same comparison logic, and applying the same uncertainty language over time. This alone would make repeated detection outputs easier to interpret and compare.

Implementation also requires clear roles. Laboratories generate and document the measurements. Program analysts integrate the chemical and biological evidence and record major comparability limits. Managers or regulators use the tiered output to decide what follow-up is needed. In this sense, the framework becomes operational only when standardized detection outputs are translated into a repeatable reporting and decision routine.

## 7 Remaining gaps and pathway toward consensus standards

Broader adoption of this framework depends on three linked but distinct gaps: methodological standardization gaps, interpretive tier-classification gaps, and implementation gaps.

Methodological gaps concern how antibiotic and ARG/ARB data are generated, detected, annotated, and made comparable. On the antibiotic side, recent standardization studies have already moved beyond a generic call for better chemistry and started to define analytical performance expectations, quantify the effect of calibration strategy, and identify reporting choices that matter for surveillance rather than only for single studies (Fabregat-Safont et al., 2023; Magee et al., 2023; Thurner and Alatraktchi, 2023; Byrnes et al., 2025). Those studies provide an important foundation, but they do not by themselves resolve the central claim of this review: chemical and biological standardization must ultimately be designed for joint interpretation rather than as two parallel reporting exercises. On the ARG/ARB side, prior standardization papers and framework reviews have already emphasized core targets, denominators, comparability qualifiers, and the minimum metadata needed for repeated interpretation across sites and time (Liguori et al., 2022; Nava et al., 2022; de Beltran Heredia et al., 2025). This review builds directly on that literature, but adds a further requirement: biological standardization is still incomplete if the selected targets and reporting basis cannot be read against the antibiotic exposure profile developed in Sections 3 and 4. A second priority, no less important than denominator and metadata standardization, is convergence on ARG definitions, reference databases, and annotation rules (Papp and Solymosi, 2022). As noted in Section 4.2, this is not only a future harmonization issue: reference-database choice and annotation thresholds already shape current sequencing-based ARG detection outputs and determine how comparable those outputs are across programs. CARD, ResFinder, NDARO or AMRFinder, and MEGARes were built for different analytical purposes and do not classify resistance determinants in exactly the same way. As a result, the same sequence data can yield different ARG abundance profiles and category assignments depending on the reference system used, meaning that database choice can directly affect downstream comparison and tier-relevant interpretation (Alcock et al., 2020; Doster et al., 2020; Feldgarden et al., 2022). Near-term progress therefore requires both transparent reporting of database version, annotation thresholds, and cross-database translation choices, and more explicit efforts to harmonize ARG definitions and classification rules themselves rather than treating database choice as a minor reporting detail (Papp and Solymosi, 2022; Feldgarden et al., 2022).

Interpretive tier-classification gaps concern how combined exposure and ARG/ARB evidence is translated into tiers of concern and uncertainty statements. Experience from other monitoring fields suggests that tiered categories become useful only after they are stress-tested across settings and linked to predefined response logic, not when they remain purely descriptive (Maruya et al., 2016; Brosky et al., 2024). For this framework, the practical questions are whether the same tier remains stable when temporal coverage is short, when exposure and resistance signals are only partly aligned, or when comparability is stronger within programs than across them (Chau et al., 2022; Flach et al., 2021). Comparable work in wastewater public-health surveillance and in tiered contaminant-monitoring frameworks points in the same direction: categories and uncertainty language are most credible when they are tied to explicit assumptions about data coverage, comparator choice, and intended action (Maruya et al., 2016; Brosky et al., 2024; McClary-Gutierrez et al., 2021). The same principle should apply here. Uncertainty statements should explain why a tier was assigned and how much weight it should carry, not simply add another label.

Implementation gaps concern the handoff from monitoring output to reporting summary to follow-up recommendation. This is not simply a laboratory problem. Integrated surveillance reviews, reporting-guideline efforts, and wastewater public-health frameworks all show that useful systems depend on stable core fields, a consistent summary format, and a defined point at which interpretation becomes an operational recommendation (Babu Rajendran et al., 2023; Delpy et al., 2024; Lappan et al., 2024). For antibiotic-ARG surveillance, that means the reporting template in Section 6 should be treated as part of the surveillance design rather than as an afterthought. Consensus is therefore more likely to emerge first around shared reporting fields, tier wording, uncertainty statements, and escalation logic than around fully unified analytical methods across all settings.

A realistic pathway toward consensus standards should therefore be staged rather than absolute. A first stage is agreement on the minimum monitoring package and the metadata needed to interpret it. A second stage is convergence on comparison rules within the chemical and biological streams, including annotation transparency on the ARG side and declared comparison frames on the antibiotic side. A third stage is multi-program piloting of shared tier definitions, uncertainty statements, and reporting templates. A fourth stage is incorporation of those reporting and interpretation rules into routine management workflows. Just as wastewater-based epidemiology and other integrated surveillance fields have usually progressed through minimum datasets and harmonized reporting before attempting full methodological uniformity, antibiotic-ARG surveillance is likely to advance more reliably through the same sequence (Babu Rajendran et al., 2023; Lappan et al., 2024; Brosky et al., 2024; McClary-Gutierrez et al., 2021).

The framework does not need every unresolved question to be settled before it can be useful. The most practical near-term gains are likely to come from better integration of antibiotic exposure monitoring, standardized ARG/ARB reporting, and harmonized tiered interpretation, while more specialized evidence layers remain later extensions. The next step is therefore not complete uniformity, but staged progress toward surveillance that is comparison-ready and usable in decision-making.

## 8 Conclusion

Environmental detection should not stop at ARG or ARB detection alone. It becomes more useful for decision-making when antibiotic exposure monitoring, standardized biological reporting, and harmonized qualitative tiering are brought together in a single interpretive framework. Existing standardization efforts in antibiotic chemistry and ARG surveillance provide essential foundations, but they are not enough if the two evidence streams continue to be generated and interpreted in parallel.

The framework proposed here treats standardization as a workflow problem rather than a laboratory-only one. A minimum detection package, comparison-ready chemical and biological outputs, explicit uncertainty statements, and tier-linked reporting are all needed if detection results are to support prioritization, follow-up, and proportionate management responses. Its purpose is not to infer direct environmental or public-health risk from routine monitoring alone, but to improve how surveillance evidence is compared, contextualized, and used.

The immediate objective is not universal methodological uniformity across all settings, but staged convergence on shared detection elements, transparent reporting rules, and integrated interpretation of antibiotic exposure and ARG/ARB evidence.
